# Genesis-DB: a database for autonomous laboratory systems

**DOI:** 10.1093/bioadv/vbad102

**Published:** 2023-08-02

**Authors:** Gabriel K Reder, Alexander H Gower, Filip Kronström, Rushikesh Halle, Vinay Mahamuni, Amit Patel, Harshal Hayatnagarkar, Larisa N Soldatova, Ross D King

**Affiliations:** The Department of Computer Science and Engineering, Chalmers University of Technology, Gothenburg, 412 58, Sweden; The Department of Computer Science and Engineering, Chalmers University of Technology, Gothenburg, 412 58, Sweden; The Department of Computer Science and Engineering, Chalmers University of Technology, Gothenburg, 412 58, Sweden; Engineering for Research (e4r™), Thoughtworks Technologies (India) Pvt Ltd, Pune, 411006, India; Engineering for Research (e4r™), Thoughtworks Technologies (India) Pvt Ltd, Pune, 411006, India; Engineering for Research (e4r™), Thoughtworks Technologies (India) Pvt Ltd, Pune, 411006, India; Engineering for Research (e4r™), Thoughtworks Technologies (India) Pvt Ltd, Pune, 411006, India; Department of Computing, Goldsmiths, University of London, London, SE14 6AD, United Kingdom; The Department of Computer Science and Engineering, Chalmers University of Technology, Gothenburg, 412 58, Sweden; Department of Chemical Engineering and Biotechnology, University of Cambridge, Cambridge, CB3 0AS, United Kingdom; Alan Turing Institute, London, NW1 2DB, United Kingdom

## Abstract

**Summary:**

Artificial intelligence (AI)-driven laboratory automation—combining robotic labware and autonomous software agents—is a powerful trend in modern biology. We developed Genesis-DB, a database system designed to support AI-driven autonomous laboratories by providing software agents access to large quantities of structured domain information. In addition, we present a new ontology for modeling data and metadata from autonomously performed yeast microchemostat cultivations in the framework of the Genesis robot scientist system. We show an example of how Genesis-DB enables the research life cycle by modeling yeast gene regulation, guiding future hypotheses generation and design of experiments. Genesis-DB supports AI-driven discovery through automated reasoning and its design is portable, generic, and easily extensible to other AI-driven molecular biology laboratory data and beyond.

**Availability and implementation:**

Genesis-DB code and installation instructions are available at the GitHub repository https://github.com/TW-Genesis/genesis-database-system.git. The database use case demo code and data are also available through GitHub (https://github.com/TW-Genesis/genesis-database-demo.git). The ontology can be downloaded here: https://github.com/TW-Genesis/genesis-ontology/releases/download/v0.0.23/genesis.owl. The ontology term descriptions (including mappings to existing ontologies) and maintenance standard operating procedures can be found at: https://github.com/TW-Genesis/genesis-ontology.

## 1 Overview

Advances in artificial intelligence (AI) and automation are enabling increasingly data-intensive laboratory workflows and closed-loop discovery systems (Bai *et al.* 2022). Robot scientists—that couple automated lab hardware with AI systems to autonomously generate, test, and analyze scientific hypotheses for iterative knowledge discovery—are a prime example of these trends. Such a concept was introduced nearly two decades ago (King *et al.* 2004) with the robot scientist A Discovery Machine (ADAM) created to autonomously study yeast functional genomics. The robot scientist Eve (Williams *et al.* 2015) later studied drug design using a combination of logical induction, active learning, and econometric modeling. ADAM and Eve successfully made new discoveries autonomously, for example identifying triclosan as a potential anti-malarial. However, as with many automated laboratories, they suffered from the lack of adequate database platforms designed to support autonomous software-driven discovery. As automated laboratories scale up dramatically in size, speed, and demands there is urgent need for automation-focused data storage. Crucially, databases must support the advanced reasoning capabilities of AI systems deployed in automated laboratories. The advent of the next-generation robot scientist Genesis, aiming to execute thousands of closed-loop cycles of microchemostat culture experimentation in parallel while autonomously performing AI-driven reasoning over the resulting data, embodies these requirements.

Drawing on over 20 years of experience of working with robot scientists, we identified three major areas of functionality that a database system requires to address the needs of next-generation automated laboratories with advanced reasoning capabilities. While individual features may be present in some existing systems, no system has yet presented an all-in-one solution. These pillars are: (1) Machine-interpretable storage of data and metadata compatible with automated reasoning approaches; (2) easy-to-deploy storage for data with custom ontologies; and (3) consistent reproducibility backed by data schema stability across versions. We have created a database system with these attributes, Genesis-DB.

Genesis-DB is designed to be used with automated software agents. Many of today’s laboratory databases are primarily designed for human users whose usage differs significantly from the autonomous laboratory systems. For AI-based systems to function seamlessly, data must be encoded in a machine-interpretable format to obviate the need for human intervention. As demonstrated in previous ADAM and Eve studies, Semantic Web technologies provide means for robustly capturing the important aspects of new data, experimental metadata, and background knowledge (Soldatova *et al.* 2006). Resource Description Framework (RDF), XML, and binary representations of biological knowledge stored side-by-side with experimental protocols and metadata allow for closed-loop discovery via automated reasoning (Coutant *et al.* 2019). This machine-centeric approach centers around the use of ontologies to structure all data, thus giving data a strict underlying logic compatible with upstream AI inference layers.

A key feature of Genesis-DB is the ability to flexibly store and serve data given any ontology supplied by the user. The research community has developed hundreds of ontologies for biology serving different needs. For example, widely used ontologies exist for the description of biological systems and mathematical simulations thereof. To demonstrate the utility of Genesis-DB for automated laboratory applications, we created a novel ontology that robustly captures information surrounding the Genesis robot scientist hardware focused on automated small volume chemostat cultivations. Relevant information to capture included cultivation conditions (e.g. yeast strain, temperature, growth medium, and pH) and RNA sequencing transcript counts. We then exploited data stored in Genesis-DB with this new ontology to reconstruct a yeast gene regulatory network (GRN), making use of SPARQL, a query language well-suited for upstream automated reasoning applications.

Genesis-DB is built around the open-source Apache Jena framework (jena.apache.org). Notably, its containerized nature allows it to be deployed using orchestration platforms such as Kubernetes (kubernetes.io), easing automation of laborious processes such as scaling and monitoring.

Stability of stored data over iteration cycles is especially important in the automated laboratory setting. Typically, database curation and maintenance provides stable data access to human users (using conventional tools) but does not account for the ways AI-agents may access the data. Even slight changes in the underlying data structure unapparent to human users may break the bond between database and upstream software agents. The ADAM and Eve robot scientists both ultimately relied on internal copies of external databases to circumvent catastrophic system errors as a result of unstructured database updates. Ontology development practices dictate that deprecated classes are defined as obsolete rather than deleted in version updates. By making ontology usage a requirement for Genesis-DB data storage, we require users to enter into this stability-focused system of practices. Additionally, structured recording of experimental protocols and data improves repeatability and reproducibility of the results.

## 2 Implementation and results

### 2.1 Database implementation

Genesis-DB is based on Apache Jena (version 4.6.0) (jena.apache.org), a free and open-source Java framework for building Semantic Web and linked data applications. Most components are inherited from the Jena framework including a Fuseki SPARQL server, TDB2 triple store, and the ARQ SPARQL query engine. We note the possibility of future performance and feature optimization around these different layers based on application-specific requirements. All the components in Genesis-DB are containerized using Docker, enabling portable deployment in most environments.

### 2.2 A novel ontology for automated microchemostat cultivations (Genesis)

In order to demonstrate the ability of Genesis-DB to flexibly accept custom ontologies, we developed a new ontology incorporating some relevant aspects of the Genesis robot scientist setup. This ontology describes usage of automated microchemostats including the sample, experimental, and protocol metadata and RNA sequencing transcript counts. A visual overview of the domain model can be found in the demonstration GitHub repository. The data model follows the popular Ontology for Biomedical Investigations (OBI) guidelines (Bandrowski *et al.* 2016) and primarily includes three types of entities: material entities, information content entities, and process entities. This ontology was created with AI-powered automated laboratories in mind and balances the resulting design constraints while maximizing usage of terms from existing ontologies and remaining flexible for additions of future laboratory components. Entities in our ontology were mapped to those from a collection of widely used ontologies including the OBI, Information Artifact Ontology, Units Ontology, Ontology of Genes and Genomes, Prescription of Drugs Ontology, Food Ontology, Chemical Entities of Biological Interest, Environment Ontology, Phenotype And Trait Ontology, NCI Thesaurus Open Biomedical Ontologies (OBO) edition, and Basic Formal Ontology, all accessible through the OBO Foundry (Smith *et al.* 2007).

Defining this ontology required creation of new terms, for example the “cell culturing regime,” an information content entity describing a chemostat experiment as a sequence of individual regimes, each describing a set of steady-state culturing conditions. This term allows for modular re-use of such regimes in different experiments, crucial for the Genesis robot scientist project and its designs to run automated reasoning over massively parallelized microchemostat experiments. A sheet describing the ontology terms including term mapping descriptions to existing ontologies is linked in the “Availability and Implementation” section. Such custom ontologies will become ever more important to AI-powered automated laboratories as hardware, AI models, and protocols rapidly evolve. The full ontology is available as specified in the “Data and Code Availability” section. The Genesis development team will continue to update and maintain this ontology.

### 2.3 Demonstration of software agent usage of Genesis-DB for automated laboratory model improvement

To illustrate the usefulness of Genesis-DB for research driven by automated software agents, we test Genesis-DB on its ability to facilitate model improvement in an important biological research question: reconstruction of GRNs. Identifying regulatory interactions between genes under varying environmental conditions provides powerful insight into genotype–phenotype questions. We illustrate this case study with three parts: (1) reconstruction of a GRN for *Saccharomyces cerevisiae* using the hLICORN algorithm (Chebil *et al.* 2014) and targeted retrieval of RNA transcript counts; (2) exploration of the experimental variable space that produced this data to form hypotheses about gene regulatory interactions in unexplored conditions; and (3) recording hypotheses and experiment protocols required to test them in the database enabling efficient planning for an automated laboratory.

**Figure 1.**
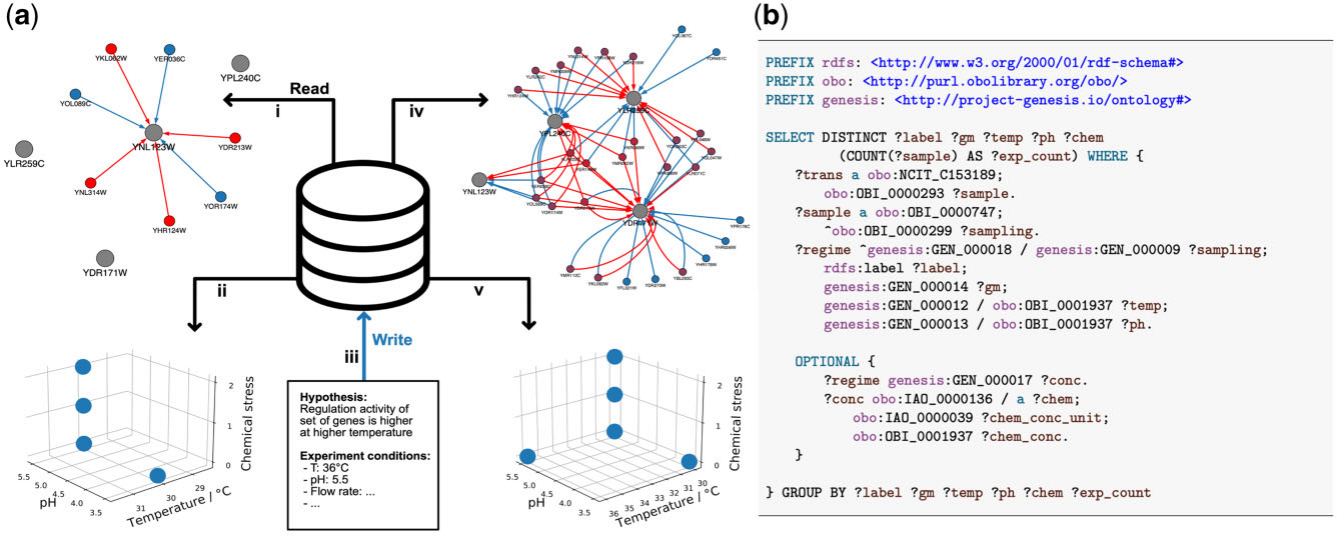
Genesis-DB a database system for autonomous laboratories. (a) Visualization of database usage from demonstration software agent utilization of experimental metadata for biological model improvement. Flow from left to right represents a cycle of model improvement. First, a GRN is reconstructed from gene counts, retrieved from the database with query (i); then the experimental conditions are retrieved (ii) and the space is visualised; (iii) a hypothesis together with the experimental procedures to test it is written to the database; then the regulatory network is recreated from data including the new high temperature experiment, retrieved using query (iv); after (v) we see that the examined conditions now also contain an experiment performed at a higher temperature. (b) SPARQL query to retrieve all experimental conditions from the database, the query used for (ii) and (v). Here, PREFIX keywords are used to specify the relevant ontologies for terms used in the query. More detail on the syntax of SPARQL is available at, e.g. Pérez *et al.* (2009).

Data from a yeast chemostat cultivation study of stress responses (Doughty *et al.* 2020) were taken and expressed in RDF by modeling it according to our novel ontology. A portion of this data corresponding to experiments conducted at 30°C was thereafter stored in Genesis-DB to simulate a first round of experimentation being conducted by an automated laboratory. SPARQL queries were then used to select data from all available experiments having associated transcriptomics measurements. Once retrieved and differential gene expressions had been calculated, the R implementation of the hLICORN algorithm [included in the CoRegNet package (Nicolle *et al.* 2015)] was used to generate a co-regulatory network. A subsection of the network is shown in the top half of Fig. 1a. Genesis-DB was then queried for the experimental conditions associated with the generation of the co-regulatory graph. The query for this data is shown in Fig. 1b. Previous work has shown that the genes YDR171W, YLR259C, and YPL240C code for heat shock proteins (Narberhaus 2002), suggesting they are more active in thermal stress conditions. The bottom left portion of Fig. 1a shows the explored experimental conditions, where the anaerobic condition and osmotic pressure due to the introduction of KCl are presented along the chemical stress axis. On seeing that there are no experiments in Genesis-DB conducted under thermal stress conditions, new experiments were specified using the ontology and “conducted” by adding 36°C data from the original dataset to Genesis-DB along with the experimental protocol and design. After completion of these experiments and addition to Genesis-DB, all transcriptomics data were once again retrieved using SPARQL queries to the database, this time including the newest experiment. The resulting improved co-regulatory graph and the experimental coverage can be seen in the transition from the left to right sides of Fig. 1a.

This cycle of data- and metadata-driven systems biology model improvement involved two software agents working on top of Genesis-DB. One is the informatic agent running hLICORN to create co-regulatory networks based on data available in Genesis-DB. The second is the metadata and hypothesis agent using Genesis-DB to explore experimental condition space and order new rounds of experiments. Both agents may interact to read from and write to Genesis-DB using SPARQL.

## 3 Discussion

In this article, we presented Genesis-DB: a database suitable for AI-driven automated laboratories that incorporates lessons learned from more than two decades of experience. We demonstrate the usage of this system by creating a novel ontology for automated microchemostat yeast cultivation and running a simulated automated laboratory cycle of multiple of rounds of experimental data collection, analysis, and model improvement.

The example provided here involves simple implementations of software “agents” as scripts run by the human user without an automated orchestration system. For example, the informatic and hypothesis agents here are, respectively, human-written data processing and visualization scripts. Interaction with Genesis-DB is programmatic; such software interaction may be triggered by a user, or by a higher level orchestration system. The degree of software autonomy and agent logic will vary with specific use-case. For the Genesis robot scientist project, Genesis-DB is intended to function in an autonomous laboratory where its primary function is to store experimental data and metadata in a manner that suits the needs of a robot scientist environment. In this case, upstream software agents are expected to interact with external data sources to derive conclusions such as those in the example suggesting increased activity in thermal stress conditions for the genes YDR171W, YLR259C, and YPL240C. We note that the programmatic interface of Genesis-DB enables different modes of application depending on the balance between software autonomy and human usage, which will differ for each laboratory and their applications.

SPARQL-driven data access provides a powerful framework for automated applications of formal logical reasoning (Arenas and Pérez 2011). SPARQL query construction can be decoupled from the minutiae of underlying data storage since each action in the query is linked explicitly to an ontology term. Translation from intent to query and from query to intent is straightforward due to the semantic meaning imparted by ontologies on which the query is based. As such, query construction itself may be automated using query methods available in popular languages including Python, C++, and R. While large language models (LLMs) have demonstrated impressive power for natural language interaction with databases, the query execution accuracy currently remains unacceptably high for scientific research involving automated interaction with the physical laboratory (Rajkumar *et al.* 2022). LLMs pose an exciting possibility for use as upstream agents as their cost (physical and computational) decreases, and a future research question is how they can be trained to interact with our database through SPARQL queries. Ongoing improvements to Genesis-DB include resource distribution, parallelization, and query optimization for improvements to scale performance with growing automated laboratory needs.
